# Thermal and Mechanical Properties of Silica-Reinforced SBR/NR/NBR Rubber Composites for Boot Tread Production

**DOI:** 10.3390/polym18030361

**Published:** 2026-01-29

**Authors:** Gordana Marković, Suzana Cakić, Marija Krstić, Marija Milenković, Slavica Porobić Katnić, Suzana Samaržija-Jovanović, Duška Kleut, Vojislav Jovanović, Marija Gizdavic-Nikolaidis, Milena Marinović-Cincović

**Affiliations:** 1 Tigar, 18300 Pirot, Serbia; gordana11markovic@gmail.com; 2 Faculty of Technology, University of Niš, 16000 Leskovac, Serbia; suzana_cakic@yahoo.com; 3 Faculty of Technology, University of Novi Sad, 21000 Novi Sad, Serbia; mkostic607@gmail.com; 4 Vinča Institute of Nuclear Sciences—National Institute of the Republic of Serbia, University of Belgrade, 11000 Belgrade, Serbia; marija.kojic@vin.bg.ac.rs (M.M.); slavic.porobic@vin.bg.ac.rs (S.P.K.); duskakleut@gmail.com (D.K.); 5 Faculty of Sciences and Mathematics, University of Priština-Kosovska Mitrovica, 38220 Kosovska Mitrovica, Serbia; suzana.samarzija@pr.ac.rs (S.S.-J.); vojislav.jovanovic@pr.ac.rs (V.J.); 6 Department of Molecular Medicine and Pathology, School of Medical Sciences, The University of Auckland, Auckland 1023, New Zealand; m.gizdavic@auckland.ac.nz

**Keywords:** rubber blend, mechanical properties, thermal properties, AFM and FTIR analysis, boot tread rubber-based compounds

## Abstract

This study investigated the influence of acrylonitrile-butadiene rubber (NBR) at 5 and 15 phr on the properties of silica-filled styrene-butadiene /polyisoprene (SBR/NR) rubber blends intended for boot tread production. Fourier Transform Infrared Spectroscopy evaluated the performance of the resulting SBR/NR/NBR composites with Attenuated Total Reflectance (FTIR-ATR), which confirmed interactions between the rubber matrix and the silica filler. In addition, changes in thermal and mechanical properties, as well as cross-linking parameters, were systematically examined. Differential scanning calorimetry (DSC) and thermogravimetric analysis (TGA) are used to provide a comprehensive understanding of the structural, thermal, and mechanical behavior of silica-reinforced SBR/NR/NBR composites. The rheological characteristics of the tested composites were examined as a function of the mixture ratio. Atomic force microscopy (AFM) revealed variations in the sample’s surface roughness and morphology with varying rubber blend ratios. The findings confirmed that incorporating NBR improves filler dispersion, increases cross-link density, and enhances mechanical properties, including hardness and tensile strength, while also influencing thermal stability and curing behavior. The results suggest the potential of these composites for reliable, efficient sole manufacturing in the footwear industry, where durability, strength, and processability are critical requirements.

## 1. Introduction

Functional elastomeric materials based on cross-linked polymers and nanostructured fillers represent systems of considerable economic and environmental significance. Over the past decades, intensive scientific and industrial efforts have been directed toward advancing research and commercialization in the field of rubber blends. To achieve rubber materials with superior performance and broad applicability, different types of rubber are blended to combine the specific advantages of each component. Such blending strategies are essential for enhancing mechanical properties, optimizing processing behavior, and improving overall economic viability.

Numerous researchers have extensively studied the design of functional elastomers and fillers to develop composite materials suitable for the production of environmentally friendly boot treads [1,2,3,4]. An eco-friendly boot tread is generally a system composed of components with distinct chemical properties, where an appropriate combination of these elements yields a composite with enhanced characteristics, such as wear resistance, comfort, and waterproofing. The creation of new composite materials through polymer blending of elastomeric systems for commercial applications is considered both efficient and cost-effective. A crucial factor for the practical usability of polymer composites is their compatibility, since blends of two incompatible elastomers in a crosslinked system typically exhibit reduced rheological and mechanical performance [5]. In multiphase systems, the properties of individual phases can be tailored or modified through appropriate design, thereby influencing the overall performance of the composite. Recent advances in the rubber and footwear industry have increasingly focused on the strategic blending of existing polymers, aiming not only to improve the properties of conventional elastomeric materials but also to develop entirely new classes of composites with superior functionality [6]. Such approaches highlight the importance of nanostructured fillers and polymer blends in achieving sustainable, high-performance materials for practical applications.

Styrene-butadiene rubber (SBR) is one of the components of elastomeric blends in the production of car tires, wires, and cables. SBR is not smooth and homogeneous, with high elongation and fracture values, but has a very low modulus of elasticity and durability. By adding antioxidants, accelerators, softeners, and fillers, some mechanical properties are improved. Nitrile rubber (NBR) has high oil and chemical resistance [7]. Natural rubber (NR) has good flexibility, but its limited application is due to its poor resistance to external factors, such as oil and ozone, as well as to thermal aging. Elastomer mixtures are used to improve the properties of natural rubbers (thermal aging resistance, oil resistance, compression, ozone resistance, flexibility) [8]. NR and SBR are commonly blended in different ratios to reduce production costs while improving mechanical and functional properties. Successful blending of these materials can therefore yield composites with a wide range of applications in the rubber industry. To further optimize performance, incorporating compatibilizers—such as NBR—is a widely adopted strategy. By enhancing interfacial adhesion between polymer phases and fillers, NBR improves blend compatibility, thereby contributing to better processability, balanced mechanical properties, and long-term durability of reinforced rubber compounds.

Fillers, as integral components of rubber compounds, play a decisive role in determining both the properties and the overall cost of the final product. By modifying the internal structure of the polymer matrix, fillers can significantly influence mechanical strength, elasticity, durability, and even processing behavior. In recent years, fillers containing nanoparticles have attracted particular attention due to their high potential to enhance the mechanical, chemical, and physical performance of polymer blends [9]. Their nanoscale dimensions enable stronger interfacial interactions with the polymer chains, leading to improved dispersion, enhanced reinforcement efficiency, and multifunctional properties. The particle size, i.e., the reduced dimension, together with the high surface-to-volume ratio of nanofillers, plays a crucial role in enhancing the mechanical and thermal properties of composite materials [10]. Consequently, nanoparticle-based fillers are increasingly recognized as key materials in the design of advanced elastomeric composites with tailored properties for diverse industrial applications.

Nanocomposite materials, often referred to as organic–inorganic hybrids, enable the effective integration of organic polymers with inorganic components. This synergy results in lightweight, flexible materials that exhibit high strength, excellent compressive performance, enhanced thermal stability, and superior chemical resistance [11]. Furthermore, by carefully tailoring the nanofiller structure and dispersion within the polymer matrix, it is possible to achieve advanced functional properties, including improved wear resistance, reduced flammability, optimized electrical and electronic performance, controlled gas permeability, and enhanced heat resistance [12]. Such multifunctional characteristics make nanocomposites highly attractive for a wide range of industrial and technological applications.

Several research groups have examined SBR/NR mixtures [13,14,15,16,17] and their physical properties, including wear resistance, flexural, tensile, and compressive strengths. Jovanović et al. [18] showed that carbon blacks influence changes in physical-mechanical properties before and after thermal aging. Using standard thermal analysis methods, a group of researchers [19] investigated heat and heat-resistance reactions under non-isothermal conditions. Meanwhile, a group of scientists, Mantia et al. [20] studied molecular chain reactions of composite systems at higher temperatures using FTIR.

This study aimed to investigate the influence of acrylonitrile-butadiene rubber (NBR) at 5 and 15 phr on the properties of silica-filled SBR/NR blend composites. The study characterized both SBR/NR and SBR/NR/NBR blends with varying rubber compositions, specifically SBR/NR without NBR (0 phr) and SBR/NR/NBR with 5 and 15 phr of NBR. All rubber compounds are reinforced with silicon dioxide (SiO_2_) as a filler to develop rubber composites suitable for making boot treads with appropriate mechanical and thermal properties.

The significant novelty of this research lies in the formulation of rubber composites specifically tailored for boot tread applications. Incorporating NBR into silica-filled SBR/NR blends has been shown to improve the balance between mechanical and thermal properties, while simultaneously enhancing key performance indicators such as durability, flexibility, and processability. These findings underline the role of NBR as an effective compatibilizer, capable of modifying filler–matrix interactions and increasing crosslink density, thereby yielding composites with superior structural integrity. Taken together, the results highlight the potential of these materials to serve as reliable alternatives in the footwear industry, meeting the demanding requirements of tread performance and longevity.

## 2. Materials and Methods

### 2.1. Materials

The following materials were used for this study: Polyisoprene rubber (NR), Mooney viscosity M_L_ (1 + 4) 100 °C = 60 ± 5 was supplied by Malaysia with 10 phr; Emulsion styrene-butadiene rubber, SBR (HIPREN EM 1502 T) produced in HIP Pančevo (Serbia), Mooney viscosity M_L_ (1 + 4) 100 °C = 51 (ρ = 0.94 g/cm^3^), (23.5% bound stirene) with 90 phr; Butadiene acrylonitrile rubber (NBR), Mooney viscosity M_L_ (1 + 4) 100 °C = 45, (ρ = 0.98 g/cm^3^), (33% acrylonitrile) with 5 and 15 phr, produced in Italy. The proportions of the SBR/NR and SBR/NR/NBR blends were always adjusted so that the total rubber content amounted to 100 phr. 15 nm silica (ρ = 1.95 g/cm^3^) was used as a filler (Degussa, Germany). The silica content was constant (46 phr) across all tested rubber blends. The tread of rubber footwear is produced from appropriate elastomers, combined with standard sulfur cross-linking systems and additives such as plasticizers and antioxidants, which provide wear resistance and durability. The detailed original formulation used to compound the rubber blends cannot be disclosed for confidentiality reasons, as it is protected under AD Tigar’s trade secret policy in Pirot, Serbia.

The compounds were processed on a laboratory mixing mill with roller dimensions of 400 × 150 mm, at a roller speed ratio of n1/n2 = 28/22 and a roller temperature of 40–50 °C. SBR and NBR were masticated for 3 min and then blended with NR. Homogenization of the rubber mixture lasted 7 min, after which the other ingredients were incorporated.

### 2.2. Cure Characteristic

The cure characteristics (scorch time t_s2_, cure time t_c90_, minimum M_L_, and maximum torque M_H_) of rubber compounds were determined in accordance with ASTM method D-2084 using a Monsanto rheometer (ODR model 4308 from Zwick, Ulm, Germany) at 160 °C. Vulcanization experiments were performed in an electrically heated laboratory hydraulic press at 160 °C under about 4 MPa pressure. The tested rubber plate samples were 300 × 300 mm. All samples were conditioned for 24 h at 25 °C before testing.

Using Equation (1), the values for the cure rate index (CRI) were calculated:

(1) $$CRI=\frac{1}{t_{C90}-t_{s2}}$$

### 2.3. Swelling Experiments

For swelling ratio assessments, vulcanized rubber sheets (2 mm thick) were cut into 20 mm × 5 mm pieces. These samples were then dried in a vacuum oven at 60 °C for 24 h to eliminate internal moisture and gases, preserving the material’s integrity. To prevent aging from external factors like light and heat, the dried samples were stored at 0 °C. Before testing, the specimens were acclimatized to room temperature for over 4 h. Swelling measurements, conducted both temporally and thermally, aimed to ascertain the rate and temperature dependency of swelling. Equilibrium in swelling for all solvents was observed after 24 h. The solvents used were toluene. For each experiment, three dried specimens were placed in individual 10 mL vials containing the solvent. These vials were then immersed in a temperature-controlled water bath at the specified temperature for a set duration. To mitigate external factors like light and oxygen, which could accelerate swelling, the water tank was covered during immersion. The final swelling ratio was determined by averaging the results from the three specimens. The degree of swelling Q is calculated as:

(2) $$Q\;=\frac{w-w_{o}}{w_{o}}\text{·}\frac{\rho_{r}}{\rho_{s}}$$

where w and w_o_ are the masses of the sample before and after swelling; ρ_r_ and ρ_s_ are the densities of the crosslinked materials and solvents, respectively.

### 2.4. Mechanical Properties

The tensile properties of the nanoblends (TS and Eb) were tested according to ASTM D-412. A Zvick-1425 machine (ZwickRoell GmbH & Co., KG, Ulm, Germany) with a separation rate of 500 mm/min and a temperature of 25 °C was used for testing. The mean value of the three examined samples was taken. The hardness of the samples was determined according to the ASTM D-2240 standard test method. A mean of three measurements was taken. The specific gravity was determined using Equation (3):

(3) $$\rho =\left(\frac{W_{a}}{W_{a}-W_{w}}\right)\cdot \rho_{w}$$

*W_a_*—sample weight in air, *W_w_*—sample weight in water, *ρ_w_*—water density.

Abrasion resistance (ASTM 2228) as determined according to Equation (4) and expressed as volume loss in mm^3^:

(4) $$A=\frac{\left(G_{0}-G\right)\cdot 200}{\rho }\cdot K$$

*G*_0_—weight of rubber spacement before abrasion (g); *G*—weight of rubber spacement after abrasion (g); *ρ*—rubber specific gravity (g/cm^3^); *K*—abrasive sharpness, relative number of abrasive 0.180–0.200 (standard tests determine its values); 200—factor for cm^3^ translates in mm^3^.

### 2.5. Thermogravimetric Analysis

Thermogravimetric analysis of silica-reinforced SBR/NR nanocomposite without NBR and SBR/NR/NBR with 5 and 15 phr of NBR was investigated by the TGA, DTG, and DTA techniques, using a Setaram Setsys Evolution 1750 instrument (Setaram Instrumentation, KEP Technologies, Caluire-et-Cuire, France). The samples were heated from 30 to 800 °C at a flow rate of φ = 20 cm^3^/min under an argon (Ar) atmosphere, with a heating rate of 10 °C/min. The average mass of the samples’ nanocomposites was about 5 mg.

### 2.6. DSC Analysis

The glass transition temperature (T_g_) was measured by DSC (DuPont 910 differential scanning calorimeter, DuPont Instruments, Wilmington, DE, USA). The experiments were performed in the range from −70 to −10 °C (heating rate 10 °C/min) in an inert atmosphere (N_2_). The glass transition temperature (T_g_) was determined as the midpoint of the heat capacity change observed in the DSC thermogram, corresponding to the temperature at which half of the step in the specific heat capacity is completed, and thus represents the average onset of cooperative segmental mobility in the amorphous phase

### 2.7. FTIR Measurements

FTIR measurements were conducted using a Perkin Elmer Spectrometer with a Smart iTX ATR accessory (PerkinElmer, Inc., Shelton, CT, USA). The sample thickness ranged from 0.3 to 0.4 mm. For FTIR-ATR measurements, samples were prepared between Teflon films at 163 °C using the compressor mold method. FTIR spectra were recorded in the wavenumber range 400–4000 cm^−1^, with a resolution of 4 cm^−1^ and 64 scans per spectrum.

### 2.8. AFM Measurements

Atomic force microscopy (AFM) measurements were performed on a Quesant microscope (Quesant Instrument Corporation, Agoura Hills, CA, USA) operating in tapping mode in the air at room temperature. A thin slice of the specimen was glued to the holder using double-sided adhesive tape. Silicon tips (purchased from Nano and More) with a constant force of 40 N/m were used. AFM images were analyzed using Gwyddion software (version 2.53).

## 3. Results and Discussions

### 3.1. Cure Characteristics of Rubber Blend Nanocomposites

The cure characteristics of the compounds were investigated at 160 °C. Scorch time (ts_2_), optimum cure time (tc_90_), minimum-M_L_ and maximum-M_H_ torque, the difference that exists between maximum and minimum torque-ΔM (strength index), and cross-linking rate index (CRI) are listed in Table 1.

ΔM, defined as the difference between M_H_ and M_L_, is primarily governed by the quantity of free curatives in the compound and can be regarded as an indirect indicator of the cross-linking density of the rubber blend. For the silica-filled compounds, the ΔM values of the SBR/NR/NBR blends are higher than those of the blends without NBR by more than 6%. This is a noteworthy result, as it indicates that NBR adsorbed on the silica surface prevents silica from binding the curatives. Since ΔM primarily depends on the amount of free curatives in the compound, this finding highlights NBR’s role in modifying curing behavior. This observed increase in ΔM can be directly attributed to specific interactions at the silica surface, which govern the adsorption of curatives and compatibilizers. Silica possesses numerous hydroxyl groups on its surface, leading to strong filler–filler interactions and the adsorption of polar materials via hydrogen bonding. Consequently, filler dispersion in silica-filled rubber compounds is generally poorer than in carbon-filled systems. The polar surface of silica forms hydrogen bonds with polar materials in the rubber matrix, and, due to its acidic nature, it interacts particularly strongly with basic compounds. Used a cure accelerator, which contains basic functional groups such as amides (RCO–NR_2_), which are readily adsorbed onto the silica surface. This adsorption of curatives leads to delayed scorch time and reduced delta torque in silica-filled rubber compounds. Acrylonitrile-butadiene rubber (NBR), however, contains nitrile groups (–CN), which are also basic and can form hydrogen bonds with silica, thereby altering adsorption dynamics and improving curing efficiency [21,22,23].

El-Sabbagh et al. [24] found that cross-link densities and chain entanglements determine M_H_ values. For silica-filled compounds, t_c90_ increased at 15 phr NBR. This may be due to a reduction in curative adsorption and to the activation of the zinc complexed by NBR. The t_s2_’s of the silica-filled compounds containing NBR of 5 and 15 phr are slightly slower than that of the compound without NBR. This implies that for silica-filled SBR/NR compounds, adding NBR increases the cure rate without accelerating scorch time when the NBR content is 15 phr or less.

The curing properties of composites based on SBR/NR/NBR boot tread compounds reinforced with silica have shown better performance than those of SBR/NR compounds. Silica, as a filler with its surface silanol groups, acts as an accelerator [25]. Therefore, the values for M_H_ and ΔM occur as a consequence of the creation of a chemical and physical network. Applying the mixing law, the mixture is expected to have a mean specific gravity between 0.98 g/cm^3^ for NBR, 0.94 g/cm^3^ for SBR, and 0.92 g/cm^3^ for NR. That is, increasing the NBR content in the mixture affects achieving a non-extreme variation in specific weights.

### 3.2. Mechanical Properties of Rubber Blend Nanocomposites

**Figure 1:**
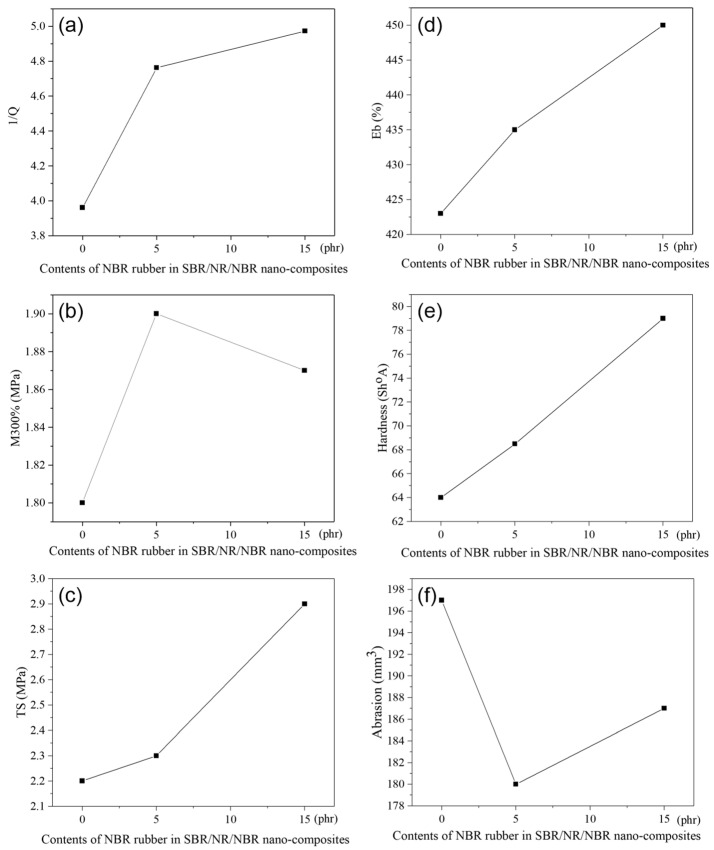
Mechanical properties of SBR/NR/NBR boot tread rubber blend nanocomposites reinforced with silica: (**a**) Rubber resistance to swelling; (**b**) M300%; (**c**) TS; (**d**) Eb; (**e**) Hardness, and (**f**) Abrasion.

The dependence of M300%, TS, hardness, E_b_, abrasion resistance, and Q on the blend compositions is shown in Figure 1a–f.

The degree of swelling, Q, is presented in the literature as its reciprocal value, 1/Q, and is, in fact, a measure of the degree of cross-linking or resistance of the rubber to swelling. The higher this value, the more resistant the rubber is to swelling (i.e., the more crosslinked and denser the network). From Figure 1a, it can be seen that this value increases with the addition of NBR to the SBR/NR/NBR nanocomposite. M300%, hardness, TS, and Eb values increase with the addition of NBR, which can be attributed to an increase in cross-link density in the rubber-based systems [26]. Therefore, NBR plays a significant role in SBR/NR/NBR blends, improving overall mechanical performance. This effect becomes particularly evident in silica-filled vulcanizates, where the hardness, modulus, and tensile strength of the compounds containing NBR are higher than those of the vulcanizates without NBR. Conversely, the Eb of the NBR-containing vulcanizates is lower, reflecting a higher cross-link density than in systems without NBR. In this way, incorporating NBR not only improves reinforcement but also alters the balance between strength and elasticity, underscoring its importance in tailoring the properties of silica-filled rubber composites. NBR improves silica dispersion, likely via hydrogen bonding between silica hydroxyl groups and NBR nitrile groups. This adsorption reduces filler–filler interactions, enabling better dispersion and enhanced composite performance [27].

Hardness, M300%, TS, Eb, and abrasion resistance were measured to better understand the physico-mechanical properties of the nanocomposite-reinforced silica-blended boot tread rubber. As previously reported [28,29,30], the addition of fillers significantly improves the mechanical, thermal, and other properties of polymeric materials.

The effects of fillers on the polymer matrix depend on their nature, size, area, structure, shape, compatibility, and specific interactions with the matrix. One of the important properties that determines the mechanical properties of rubber nanocomposites is the surface activity of the filler, which determines the polymer-filler interaction [31].

Abrasion resistance in rubber compounds is governed by a complex interplay between modulus, hardness, and cross-link density [12,32]. Generally, higher modulus and increased cross-link density contribute to improved wear resistance, as the material becomes better able to withstand mechanical stresses and surface damage. This trend was observed in our NBR-containing blends, where abrasion loss values were consistently lower than in NBR-free blends (Figure 1f). The improved performance can be attributed to the higher cross-link density of NBR-containing vulcanizates, which enhances their mechanical integrity.

However, at elevated NBR content (15 phr), a slight decrease in abrasion resistance was recorded. This behavior can be explained by the reduced toughness of the system: although hardness and modulus are increased, excessive stiffness limits the material’s ability to dissipate mechanical stresses through elastic deformation. As a result, localized surface stress concentrations occur, leading to greater abrasion loss. Similar findings have been reported in the literature, where excessive cross-linking or high hardness may induce brittleness and compromise wear resistance despite higher modulus values [12,32].

Therefore, our results highlight that the addition of NBR to silica-filled SBR/NR blends generally improves abrasion resistance; however, an optimal balance between tensile strength, hardness, and cross-link density must be maintained. Excessive NBR content may shift this balance unfavorably, resulting in reduced abrasion performance. This observation is particularly relevant for practical applications such as shoe soles and tire treads, where abrasion loss must remain below 200 mm^3^ [33].

### 3.3. DSC Studies of Rubber Blend Nanocomposites

**Figure 2:**
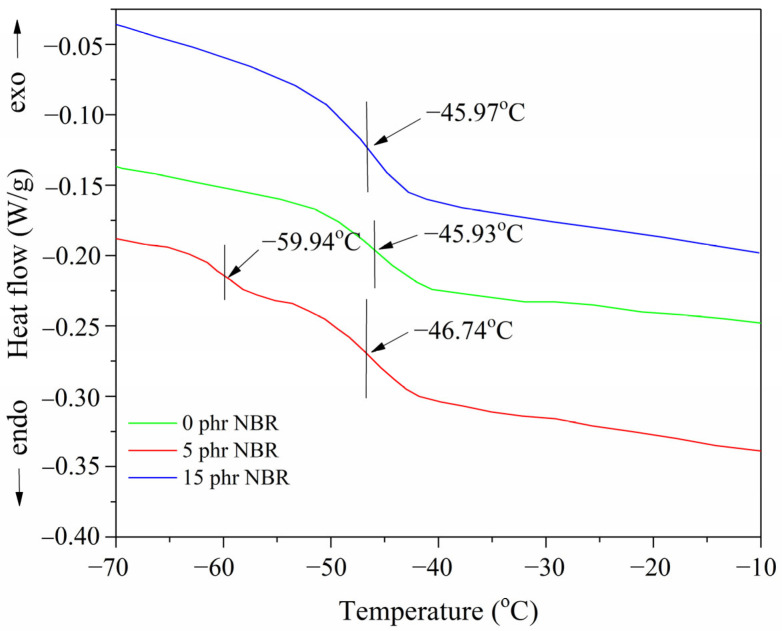
Glass transition temperature variations of SBR/NR/NBR boot tread rubber blend nanocomposites reinforced with silica.

The DSC curves of the examined nanocomposites are shown in Figure 2.

The mobility of segments within macromolecular chains of polymer nanocomposites determines the polymer’s glass transition temperature (T_g_). The values of the glass transition (T_g_) also depend on the interaction parameter of miscibility of the polymer blends that make up the investigated composite. Suppose a polymer composite is examined in which the polymer mixtures are wholly or partially immiscible. In that case, the T_g_ values remain separate or shift toward the T_g_ of one of the components of the given composite [34].

The DSC results further confirm that the presence of one or two glass transition temperatures depends on the miscibility and compositional balance of the phases. In the binary NR/SBR system (100 phr, with SBR in excess), a single T_g_ was observed at –45.93 °C, indicating partial compatibility and cooperative segmental mobility between NR and SBR. With the addition of 5 phr NBR, two distinct T_g_ values appeared (–59.94 °C and –46.74 °C), which is consistent with earlier findings that small amounts of a third component induce phase separation and heterogeneous morphology [35].

However, when the NBR content was increased to 15 phr, only one T_g_ was detected at –45.97 °C, suggesting that NBR at higher concentrations acts as a partial compatibilizer. Together with NR, it forms a cooperative mixed network in which the transitions overlap into a single dominant relaxation. These findings agree with Marzocca et al. [36], who reported that NR/SBR blends frequently exhibit a single T_g_ due to cooperative segmental mobility, whereas the introduction of a third component can either separate or merge transitions depending on its concentration. Taken together, the results and literature confirm that the occurrence of two T_g_ values does not necessarily imply complete immiscibility. Instead, it reflects the coexistence of phases with different compositional ratios, where one phase may be enriched in one polymer and the other enriched in another. At higher NBR contents, partial compatibilization is promoted, leading to a cooperative glass transition.

### 3.4. Thermogravimetric Analysis of Rubber Blend Nanocomposites

**Figure 3:**
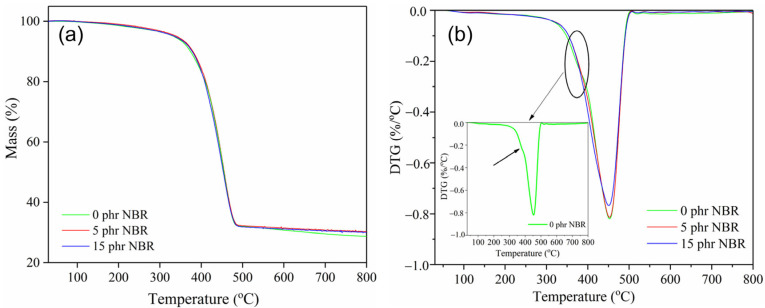
TG (**a**), DTG (**b**) curves of SBR/NR/NBR boot tread rubber blend nanocomposites reinforced with silica.

The results of the thermal stability of the tested nanocomposite materials are shown in Figure 3 and Table 2. Polymers and polymer composites are often exposed to high temperatures during application and processing, so it is crucial to assess their thermal stability [37].

**Figure 4:**
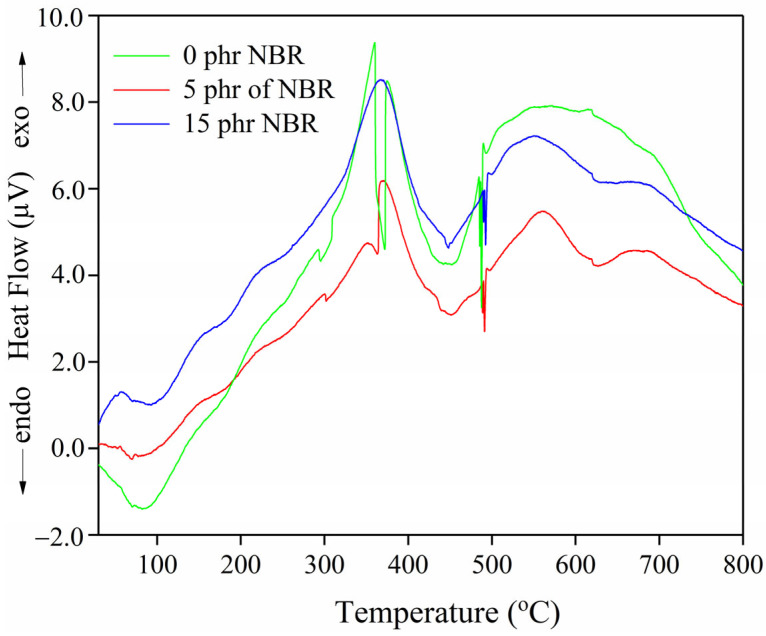
DTA curves of SBR/NR/NBR boot tread rubber blend nanocomposites reinforced with silica.

From Figure 3 and Table 2, it can be seen that the TG curves of the tested SBR/NR/NBR composites show a single-stage degradation region. As illustrated in Figure 3, the primary degradation phase (378.9–450.7 °C) is accompanied by overlapping peak interferences in the sample without NBR, indicating a two-stage thermal degradation. The overlapping peaks in the DTG curve reflect the partial superposition of degradation steps, with the decomposition of different rubber phases occurring within a similar temperature range. Although this overlap reduces the sharpness of individual DTG peaks, the mass loss data still capture the contribution of each stage. The only evidence for this is the presence of two separate exothermic peaks on the DTA curve (Figure 4). The DTG peaks correspond to the maximum rate of mass loss, while the DTA signals indicate the thermal events (endothermic or exothermic) associated with these processes. In our case, the two distinct exothermic peaks observed in the DTA curves confirm the sequential degradation stages suggested by the DTG data. Thus, the DTG overlap is consistent with the dual exothermic events in DTA, which together provide complementary evidence for a two-step thermal decomposition. The thermal behavior of the SBR/NR/NBR composites shows a clear influence of NBR content on the degradation pattern and the overall stability of the material.

The SBR/NR blend (0 phr of NBR) exhibits two distinct DTG maxima at 378.9 °C and 452.4 °C. This two-step degradation profile reflects the separate thermal decomposition of the NR and SBR phases. The first DTG peak (≈379 °C) corresponds primarily to the degradation of the NR component, which begins to decompose earlier due to its highly unsaturated *cis* 1,4 polyisoprene backbone and the presence of weaker polysulfidic and disulfidic cross-links. The second DTG maximum (≈452 °C) is associated with the more thermally stable SBR phase, whose styrene-butadiene backbone and monosulfidic cross-links degrade at higher temperatures. The presence of two well-defined exothermal peaks in the DTA curve (Figure 4) and the highest total mass loss (71.4%) indicates a heterogeneous network with limited phase compatibility. When 5 phr NBR is incorporated, the degradation pattern changes markedly: the two DTG peaks merge into a single dominant peak at 453.8 °C, accompanied by a lower total mass loss (69.8%). The transition from a two-step to a single-step degradation process suggests improved compatibility among the NR, SBR, and NBR phases and more effective co-crosslinking [38], resulting in a more uniform and thermally stable network. The DTA curve for this composition also shows an additional high-temperature endothermic event at 628.9 °C, indicating the presence of thermally resistant structures that decompose only at elevated temperatures.

At 15 phr NBR, the composite maintains a single DTG peak (450.7 °C) with the same total mass loss as the 5 phr sample (69.8%). The slight decrease in total mass loss suggests that the stabilizing effect of NBR reaches its maximum at lower concentrations, and further additions do not significantly enhance thermal resistance. Moreover, although hardness and cross-link density increase with higher NBR content, excessive stiffness may reduce the system’s toughness. This limits the material’s ability to dissipate mechanical stresses through elastic deformation, leading to localized stress concentrations and slightly higher abrasion loss, consistent with literature reports [12,32].

The DTA profiles remain within a similar temperature range (Figure 4), with characteristic endothermic and exothermic transitions corresponding to the main decomposition processes.

The low-temperature endothermic DTA peaks (78.6–92.9 °C) (Figure 4) are most likely associated with the loss of adsorbed moisture and/or weakly bound components. The slight shifts from 83.4 °C (0 phr) to 78.6 °C (5 phr) and 92.9 °C (15 phr) may be related to changes in the distribution of the filler and polymer phases, but these variations do not significantly affect thermal stability assessment. The endothermic peaks observed between 370.5 and 450.8 °C in all compositions correspond to the main decomposition processes of the polymer matrix. In comparison, the additional high-temperature endothermic peak at 628.9 °C in the 5 phr NBR blend indicates the presence of more thermally resistant structures (e.g., more stable crosslinks or filler–polymer adducts).

The exothermic DTA peaks (357.8 and 376.7 °C for 0 phr; 370.5 and 561.1 °C for 5 phr; 365.7 °C for 15 phr) reflect exothermic decomposition events and possible post-reactions occurring during thermal breakdown. The appearance of a second, higher-temperature exothermic peak (561.1 °C) in the 5 phr NBR sample suggests a more complex degradation mechanism, with the presence of thermally stable fragments that decompose only at elevated temperatures, consistent with the concept of a strengthened, more structured interphase region.

### 3.5. FTIR-ATR Spectroscopy of Rubber Blend Nanocomposites

**Figure 5:**
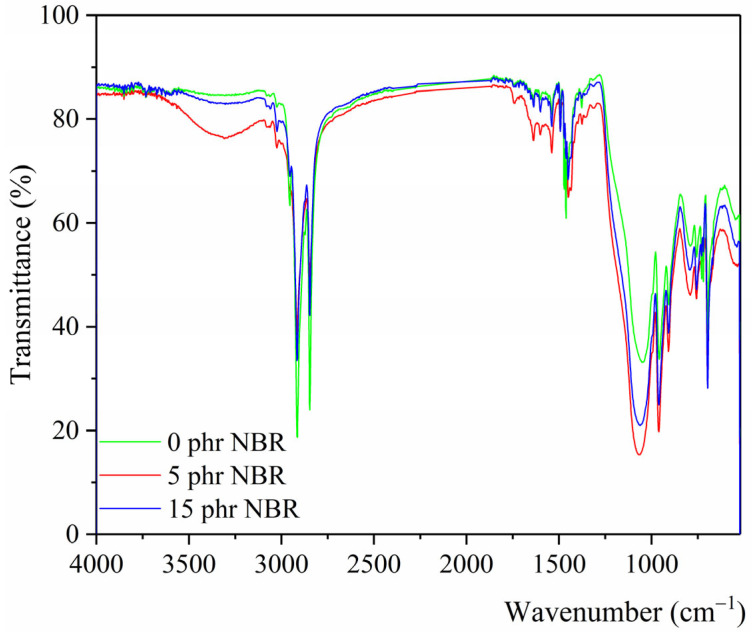
FTIR spectra of SBR/NR/NBR boot tread rubber blend nanocomposites reinforced with silica.

FTIR spectra of SBR/NR/NBR boot tread rubber blend nanocomposites with NBR (0, 5, and 15 phr) are shown in Figure 5.

In the FTIR spectrum, the broad band centered at approximately 3315 cm^−1^ is attributed mainly to hydrogen-bonded O–H stretching vibrations originating from silanol groups and adsorbed water on the SiO_2_ surface. The width of the band reflects the heterogeneous hydrogen-bonding environment typical of amorphous silica. A minor contribution from N–H stretching vibrations of curing system components cannot be excluded. In SBR, NR, and NBR samples, peaks at 2918 and 2848 cm^−1^ are assigned to CH_3_ asymmetrical and symmetrical, symmetrical C–H stretching in CH_2_, while the peak at 1464 cm^−1^ belongs to CH_2_ in-plane deformation, C-H stretching of aromatic rings. A peak at 1377 cm^−1^, attributed to CH_3_ symmetrical and CH_2_ wagging motions, is observed in NR and SBR blend nanocomposites.

The FTIR analysis demonstrates that the incorporation of NBR markedly influences the structure and organization of the interphase region between the rubber components and silica. In the NBR-free blend, the main Si–O–Si asymmetric stretching band appears at 1047 cm^−1^, whereas the addition of 5 phr NBR shifts this band to 1063 cm^−1^. This shift indicates the formation of more substantial, more specific interactions between the polar nitrile groups of NBR and the silanol groups on the SiO_2_ surface, resulting in a more constrained, structurally organized interphase region. At 15 phr NBR, the band position remains unchanged. At the same time, the increased intensity suggests that interphase saturation is achieved at lower NBR levels, with a larger fraction of silica participating in the modified interphase environment. These spectroscopic changes within the interphase region directly correlate with the macroscopic mechanical behavior: tensile strength, elongation at break, and hardness all increase with rising NBR content, while abrasion resistance improves at 15 phr NBR, although it remains below the value of the NBR-free blend. Such a property balance is particularly advantageous for rubber boot outsoles, where high strength, elasticity, and adequate abrasion resistance are simultaneously required. Our findings are consistent with previous reports on silica-reinforced NR/SBR/NBR blends, where FTIR band shifts and intensity changes were directly correlated with improved mechanical and thermal properties [39,40].

Another distinctive band of butadiene in SBR occurs at 907 cm^−1^, due to an out-of-plane C–H deformation associated with the vinyl double bond. These absorptions clearly confirm the presence of styrene and butadiene units in the SBR structure. The bonds typical of SiO_2_ (quartz, tridymite, and cristobalite) are obtained at 963 cm^−1^ for a filled SBR/NR/NBR blend, and their width depends on the chaotic state of solids [41].

The characteristic styrene absorption appears at 700 cm^−1^ and originates from a monosubstituted benzene ring, corresponding to out-of-plane C–H bending [42]. The band near 700 cm^−1^ is typically associated with vibrations of monosulfidic C–S–C linkages, which appear at higher frequencies because the C–S bond is more stable than the S–S bond. At lower frequencies, around 660 and 600 cm^−1^, bands corresponding to di- and polysulfidic crosslinks are commonly detected. It is well established that polysulfide bonds possess the lowest dissociation energy, followed by disulfide bonds, whereas monosulfide linkages are the most thermally stable. This distribution of sulfur crosslinks is directly relevant to the interpretation of TGA results, since polysulfidic bonds decompose first, followed by disulfidic bonds. At the same time, monosulfidic structures confer greater resistance to thermal degradation. Therefore, the presence and relative intensity of these FTIR bands can be directly correlated with the thermal stability of the crosslinked network observed in the TGA [43].

The FTIR spectra provide direct evidence of structural changes that correlate with the mechanical properties of the composites. The observed band shifts in the C=C stretching region and changes in the intensity of the –CH deformation bands indicate modifications to the rubber backbone upon NBR incorporation. In addition, the increased intensity of the Si–O–Si stretching band indicates stronger filler–polymer interactions, which are consistent with the higher cross-link density measured in the vulcanizates. These spectroscopic features explain the increase in modulus and hardness, as the reinforced network resists deformation more effectively. The improved abrasion resistance can also be linked to these structural changes, since stronger filler–polymer bonding reduces micro-crack initiation and wear. Thus, the FTIR results provide complementary evidence that the mechanical improvements observed in the NBR-containing blends are rooted in specific molecular and interfacial interactions, in agreement with previous studies demonstrating a correlation between FTIR spectral changes and mechanical properties in rubber composites [44,45].

### 3.6. AFM Analysis

**Figure 6:**
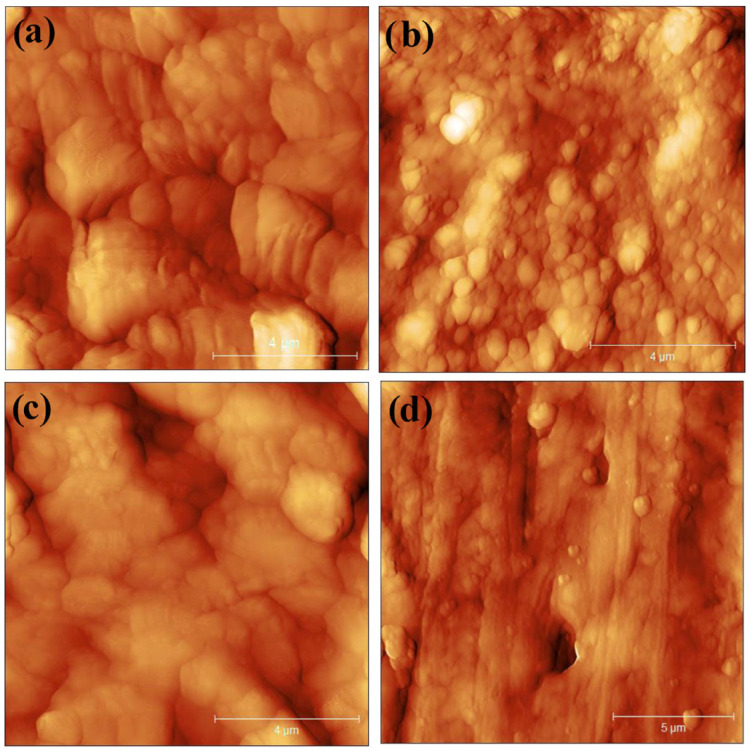
AFM images (**a**–**c**) of the three samples at 4 μm scale and (**d**) a 5 μm scale image of sample (**c**) at a different location showing the presence of SiO_2_ particles at the surface of the sample.

To investigate the NBR effect on silica dispersion, the degree of silica dispersion was examined using AFM [46]. As a measure of changes in sample morphology, the surface roughness of the samples is often calculated and compared [47]. AFM images are shown in Figure 6a–c and were taken of a representative surface morphology at a 10 × 10 μm scale of the three samples: (a) SBR/NR without NBR, (b) SBR/NR/NBR with 5 phr of NBR, and (c) SBR/NR/NBR with 15 phr of NBR. The representative images were chosen after imaging multiple points of the sample and then used for surface analysis. The surface roughness parameters calculated from these same scale images in Gwyddion are shown in Table 3. The image in Figure 6d is a 15 × 15 μm section of sample (c) showing the presence of SiO_2_ in a few random areas of the sample (c) surface.

The sample (a) SBR/NR, despite having a similar surface roughness (RMS) to sample (c) with 15 phr of NBR in the SBR/NR nanocomposite, appears to have a more varied and less flat morphology. The silica-filled compound without NBR shows very poor silica dispersion, with no visible particles. For the silica-filled compounds containing NBR, silica agglomerates are not clustered, and silica is dispersed to some extent. Some silica clusters of diameters between 100 and 500 nm are more visible in the sample with 15 phr, which can be explained by NBR adsorption onto silica and affecting the smaller RMS. The NBR adsorbed on the silica surface reduces filler–filler interactions, allowing the silica to be well dispersed in the rubber.

The microscopic analysis revealed that SiO_2_ filler particles were clearly visible in some areas of the SBR/NR/NBR nanocomposite with 15 phr of NBR, while in other blends they were not distinctly observed. This variation suggests that filler dispersion is not uniform across all systems and may depend strongly on the rubber blend composition. The presence of NBR appears to play a crucial role in modifying filler–matrix interactions. As discussed earlier, the adsorption of NBR onto the silica surface through hydrogen bonding reduces filler–filler interactions, thereby promoting better dispersion. Improved dispersion of silica enhances mechanical properties, such as higher modulus and tensile strength, but may also reduce elongation at break due to increased cross-link density.

These findings highlight the importance of compatibilizers in silica-filled systems. While silica inherently tends to form strong filler–filler networks due to its hydroxyl-rich surface, the introduction of NBR disrupts these interactions. It facilitates a more homogeneous distribution of filler particles. However, the observed differences between samples also indicate that filler dispersion may vary across regions within the same composite, suggesting that further imaging across multiple layers and depths is necessary to fully characterize the distribution. Overall, the results confirm that NBR not only improves silica dispersion but also significantly influences the balance between reinforcement and elasticity in SBR/NR/NBR composites, making it a key factor in tailoring properties for eco-friendly boot tread applications.

## 4. Conclusions

The combined FTIR, AFM, TGA, DSC, and mechanical results consistently demonstrate that the incorporation of NBR promotes the formation of a more stable and homogeneous cross-linked structure in the silica-reinforced SBR/NR system. The shift and intensification of the Si–O–Si band indicate stronger interphase interactions, supported by AFM-observed changes in surface morphology. TGA results confirm enhanced thermal stability at 5–15 phr NBR. The DSC analysis of NR/SBR/NBR blends confirms that the SBR phase remains dominant across all formulations. Still, the amount of NBR incorporated strongly influences the glass transition behavior of the blends. These findings highlight NBR’s dual role as both a phase-separating and compatibilizing component, depending on its concentration, and provide valuable insight into tailoring the morphology and thermal properties of ternary rubber systems for industrial applications.

The mechanical tests further support this interpretation: tensile strength, elongation at break, and hardness all increase with higher NBR content, while abrasion resistance improves at 5 phr but partially deteriorates at 15 phr. For rubber boot outsole applications, where high abrasion resistance is essential, the optimal balance of properties is achieved at lower NBR levels (around 5 phr). Higher NBR contents may enhance mechanical strength, but this comes at the expense of wear resistance.

Overall, the results show that incorporating NBR at 5–15 phr is an effective strategy for tuning the structural and functional performance of these composites, with careful adjustment required depending on the specific demands of the final application.

## Figures and Tables

**Table 1:** Curing characteristics of SBR/NR and SBR/NR/NBR boot tread rubber blend nanocomposites reinforced with silica.

NBR Content (phr)	Blend Compositions	M_L_ (dNm)	M_H_ (dNm)	ΔM (dNm)	t_c90_ (min)	t_s2_ (min)	CRI (min^−1^)	ρ (g/cm^3^)
0	SBR/NR/NBR	12.71	47.41	34.70	2.03	0.94	0.92	1.15
5	SBR/NR/NBR	12.50	51.88	39.38	1.86	0.83	0.97	1.16
15	SBR/NR/NBR	13.31	48.70	35.39	2.13	0.93	0.83	1.16

**Table 2:** Thermal parameters of SBR/NR/NBR boot tread rubber blend nanocomposites reinforced with silica.

NBR Content (phr)	Blend Compositions	DTG Peak Values (°C)	Mass Loss (%)	Total Mass Loss (%)	DTA Endothermic Peak Values (°C)	DTA Exothermic Peak Values (°C)
0	SBR/NR	378.9 * 452.4 *	47.7 *	71.4	83.4 370.5 449.3	357.8 376.7
5	SBR/NR/NBR	453.8	48.2	69.8	78.6 450.8 628.9	370.5 561.1
15	SBR/NR/NBR	450.7	47.5	69.8	92.9 447.7	365.7

* Overlapping peaks and the corresponding total mass loss.

**Table 3:** Surface Roughness measured on AFM images in Figure 6.

Ratio of NBR (phr)	Blend Compositions	Surface Roughness/RMS (nm)
0	SBR/NR/NBR	80.56
5	SBR/NR/NBR	106.20
15	SBR/NR/NBR	78.42

## Data Availability

The original contributions presented in the study are included in the article, further inquiries can be directed to the corresponding author.

## Abbreviations

The following abbreviations are used in this manuscript:

SBR	Styrene-butadiene rubber
NR	Natural rubber
NBR	Acrylonitrile-butadiene rubber
FTIR-ATR	Fourier Transform Infrared Spectroscopy with Attenuated Total Reflectance
DSC	Differential scanning calorimetry
TGA	Thermogravimetric analysis
DTG	Differential thermogravimetric analysis
AFM	Atomic Force Microscopy
t_s2_	Scorch time
t_c90_	Optimum cure time
M_L_	Minimum torque
M_H_	Maximum torque
CRI	Cure rate index
Q	Degree of swelling
TS	Tensile strenght
Eb	Elongation at break
ρ	Specific gravity
A	Abrasion resistance
T_g_	Glass transition temperature
